# Improvement of Hand Hygiene Practices among the Healthcare Workers in a Neonatal Intensive Care Unit

**DOI:** 10.1155/2022/7688778

**Published:** 2022-06-27

**Authors:** Abdullahel Amaan, Sanjoy Kumer Dey, Khainoor Zahan

**Affiliations:** ^1^Department of Neonatology, Bangabandhu Sheikh Mujib Medical University (BSMMU), Dhaka, Bangladesh; ^2^Bangladesh National Nutrition Council (BNNC), Mohakhali, Dhaka, Bangladesh

## Abstract

**Background:**

Healthcare workers' (HCWs) hands become progressively colonized with potential pathogens during their patient care and act as a vehicle for transmission of microorganisms to other patients. Hand hygiene is undisputedly one of the most effective infection control measures. The objective of this study was to measure the hand hygiene (HH) compliance among the doctors and nurses before and after intervention. *Methodology*. This quasi-experimental (before and after) study was conducted from July 2019 to July 2020 in the neonatal intensive care unit in a tertiary hospital in Bangladesh. The doctors and nurses were observed for their compliance to HH before and after the intervention. Several group discussions were arranged, and posters on HH were attached as reminders at the workstations during the intervention period. Binary logistic regression analysis of the predictors for the outcome as HH noncompliance was performed.

**Result:**

The overall compliance to HH was significantly increased in both before (from 42.9 to 83.8%, *p*=<0.0001) and after (28.5 to 95.9%, *p*=<0.000) patient contact, in both the case of high-risk and low-risk contacts (*p*=<0.000) following the intervention. A significant reduction in the frequency of inadequate HH (20.2 to 9.7%, *p* = .000) was documented. In logistic regression analysis, compliance to HH was found more after the intervention (aOR = 13.315, 95% CI: 7.248–24.458). Similarly, being a physician (aOR = 0.012, 95% CI: 0.005–0.030) and moments after patient contact (aOR = 0.114, 95% CI: 0.049–0.261), significant positive predictors for compliance to HH were found.

**Conclusion:**

Significant improvements in HH compliance were achieved through a systemic, multidimensional intervention approach among the doctors and nurses in an intensive newborn care setting.

## 1. Background

Recognizing the merits of hand hygiene (HH), the World Health Organization (WHO) has accepted it as the single most effective measure to prevent healthcare-associated infections (HCAIs) [1]. Globally an estimated 2·5 million neonatal deaths occur each year where infection is responsible for about 1.6 million annual deaths among neonates worldwide, 99% of which take place in developing countries [2]. In Bangladesh, infection was found to be 19.9% cause of neonatal deaths [3]. From a cross-sectional study in two large public hospitals in Dhaka, the prevalence of infection among the admitted neonates was found 69.35% [4].

Infection acquired within 48 hours of hospitalization, which was absent before or at the time of admission, is designated as healthcare-associated infection (HCAI) [5]. HCAI is a common complication during care of sick newborns in neonatal units and is associated with a high case-fatality rate, prolonged hospitalization, and adverse neurological outcome, which ultimately places a significant financial burden on the healthcare system [6, 7].

Contamination of the hands of healthcare workers (HCWs) engendered by touching the patients during routine care or coming into contact with objects in the patient environment is considered to play a leading role in patient-to-patient transmission of pathogens and to be considered the main factor in the transmission of HAIs in ICUs [8]. Consequently, hand hygiene (HH) has been proved to be the most influential, easiest, and economical method in reducing HCAIs [8, 9] which was first introduced by Austrian physician Ignaz Semmelweis in 1847 [10].

Hand hygiene (HH) is a general term referring to any action of hand cleansing. The World Health Organization (WHO) recommends either the use of alcohol-based hand rubs (ABHRs) (composed of ethanol, glycerol, and hydrogen peroxide) or handwashing with soap and water for required HH. The recommendation is to wash the hands for 40–60 seconds with soap and water when hands are visibly dirty or visibly soiled with blood or other body fluids or rubbing the hands for 20–30 seconds with at least 2 ml of an ABHR solution [11].

To improve the compliance, the WHO campaign focuses on improving HH in healthcare settings globally through multidisciplinary interventions, including regular education session, structured training programs, reminders at workplaces, observation, and feedback embedded within a hospital safety climate [12].

The presence of written messages at the workplace was found to be beneficial as a successful reminder of handwashing among the HCWs [13]. Repeated education sessions and training are the most commonly followed approach to increase awareness and improve HH compliance [14].

To evaluate the HH compliance, the direct observation method has been accepted as the gold standard for monitoring the HH compliance of HCWs. The close circuit (CC) camera system also allows indirect remote observation without the Hawthorne effect (compliance rates are often artificially high when HCWs are aware that they are being observed) [8, 15].

In the neonatal intensive care unit (NICU) of Bangabandhu Sheikh Mujib Medical University (BSMMU), there is an institutional recommendation for the HCWs to ensure proper hand hygiene (HH). In spite of that, infection has been found to be the leading cause of prolonged hospital stay and mortality among the admitted newborns. This indicates that there might be laps in the strict adherence to the recommended HH practice. Thus, to reduce the magnitude of HCAI, the hand hygiene practice among the healthcare workers needs to be evaluated and strengthened.

In this study, the effect of implementation of the WHO recommended hand hygiene strategy for improving HH compliance was assessed and the etiologies of lack of compliance on hand hygiene were explored.

## 2. Methodology

This quasi-experimental (before and after intervention) study was conducted in the Department of Neonatology, BSMMU, Dhaka from July 2019 to July 2020. Doctors and nurses serving in NICU, BSMMU were included in the study, and those who were scheduled for working for <3 months and planning for a longer duration leave (e.g., maternity leave) were excluded from the study.

The HCWs were observed anonymously in a randomly selected NICU room over an hour for their HH activities. The group discussions were conducted with 3 doctors or nurses in the NICU in their prescheduled convenient time.

Hand hygiene was defined as washing hands with soap and water for 40–60 seconds or using antiseptic hand rub for 20–30 seconds. The high-risk contacts were the invasive procedures like inserting an intravenous catheter, drawing blood, endotracheal intubation, lumbar puncture, handling wound, endotracheal suctioning, and a prolonged patient contact, e.g., bag and mask ventilation [16, 17]. The low-risk contacts were brief contacts such as tactile stimulation, padding, and tube feeding [16, 17].

Emergency rescue contact is a patient contact where an immediate intervention was essential for lifesaving, e.g., tactile stimulation and bag and mask ventilation [18]. HH opportunities were as follows: before touching a patient and any aseptic procedures, after body fluid exposure, touching a patient, and touching the patient's surroundings. They were evaluated as adequately or inadequately performed or whether they were missed.

It was a 31-bed NICU (20 beds for critical care: the proper NICU and 11 for step down care) with 7 faculties, 30 resident doctors, and 29 nurses. Resident doctors and nurses work there in 24-hour and 8-hour duty shifts, respectively. About 50–60 neonates were usually admitted per month. There were HW basins with liquid soap and an electrical hand dryer in each of the rooms. Alcohol-based hand rubs were available in each of the neonatal beds.

Informed written consent was taken from each of the participants regarding the study. In the study, both the institutional and international guidelines on research ethics were strictly maintained in all aspects after the approval of the Institutional Review Board of BSMMU.

The study was divided into a baseline observation period over 6 months, an intervention period over 1 month, and a follow-up observation period over the following 6 months.

The observation was regularly conducted on both day and night by the investigator. During the morning duty hours, it was observed directly without interfering healthcare workers' activities, and during the night shifts, it was observed by the closed circuit (CC) cameras located in the NICU.

During the intervention period, several ‘group discussion sessions' were conducted. Each of the sessions was conducted with at least 3 doctors or nurses for the convenience of answering all of their queries, without hampering the routine patient care activities. Each of the participants attended at least three such sessions of the 30–60 minute duration. At the end of each session, the WHO recommended handwashing (HW) and hand rubbing (HR) technique was practically demonstrated by the investigator and feedback HW or HR actions were directly observed from each of the participants.

The ideas of the participants regarding the causes of lack of compliance to adequate HH and their opinion on the ways how to improve the condition were discussed. It was documented with prior permission and kept confidential. After the completion of all training activities, the core materials were shared through a “telegram” group and were pasted at all the strategic locations such as near wash hand sink, beside patient beds, and consultation rooms.

During the evaluation phase, the investigator again documented the HH practices among the HCWs.

### 2.1. Data Analysis

Data were analyzed by using Statistical Package for Social Sciences (SPSS) version 26 and Microsoft Excel software. All categorical variables were compared by the Chi-square test or Fisher's exact test. Binary logistic regression analysis of the predictors for the outcome as HH noncompliance was performed. A *p*-value <0.05 was considered as statistically significant. For a qualitative study, the handwritten notes were taken immediately in the group discussion sessions by the researcher for subsequent analysis.

## 3. Result

A total of 53 healthcare workers were assessed for eligibility, and 20 of them were excluded as they were intended to work in the NICU for <3 months. Finally, 33 HCWs were observed for their compliance to the recommended HH practice. Of them 14 were doctors and 19 were nurses.

A total of 340 and 361 HH opportunities were documented before and after the intervention. A total of sixty observations (28 in preintervention and 22 in the postintervention period) were excluded from analysis as they were documented in emergency rescue contacts. Ultimately, 312 preintervention and 339 postintervention patient contacts were analyzed (Table 1).

There was a significant increase in hand rubbing events (*p*=0.000243) and a significant decrease in missing HH (*p*=0.000) after the intervention (Table 2).

Use of gloves was indicated in 67.3% and 66.9% contacts in the preintervention period and the postintervention period, respectively. Of them, 48.6% contacts were not followed by prior HH in the preintervention period which was reduced to 9.24% after the intervention. The incidence of missed wearing gloves was also significantly reduced (*p*=<0.001) after the intervention (Table 3).

Significant improvement in compliance to HH was observed prior to both the high-risk and low-risk patient contacts. For both types of patient contacts, compliance to HH was improved after the intervention; high-risk: 84 of 196 (42.9%) vs. 185 of 218 (84.9%), *p* < 0.000; low-risk: 50 of 116 (43.1%) vs. 99 of 121 (81.8%), *p* < 0.001 (Table 4).

There was a significant increase in complete HH actions and a decrease in incomplete HH actions following the intervention (Table 5).

**Figure 1:**
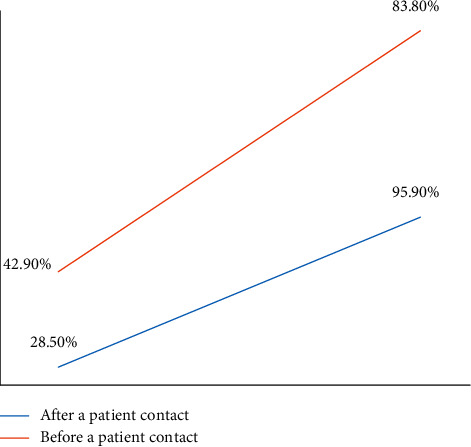
Improvement in HH compliance “before and after patient contact” following the intervention.

On the baseline observation, the proportion of HH compliance was found to be 42.9% and 28.5% before and after patient contact, respectively. A statistically significant improvement was observed both in the moment before (from 42.9 to 83.8%, *p*=<0.001) and after (28.5 to 95.9%, *p*=<0.001) the patient contact following the intervention (Figure 1).

At the baseline observation, compliance of doctors before the patient contact was found better than the nurses (84.4% vs. 11.3%), but nurses were more compliant after the patient contact (38.4% vs. 15.6%). After the intervention, compliance of HH was observed to increase in both the case of doctors (15.6 vs. 97.8%) and nurses (38.4 vs. 95.2%) (Table 6).

In logistic regression analysis, HCWs were found significantly more noncompliant to HH before intervention (aOR = 13.315, 95% CI: 7.248–24.458). Similarly, being nurse (aOR = 0.012, 95% CI: 0.005–0.030) and events before patient contact (aOR = 0.114, 95% CI: 0.049–0.261), significant determinants of compliance were found (Table 7).

### 3.1. The Result of Discussion Sessions on HH with HCWs

Each of the participants agreed that it is essential to ensure proper hand hygiene (HH) in their duty hours in the NICU to protect the admitted newborns, as well as, themselves from cross transmission of infections.

“Sometimes in a hurry, especially in the evening and night shifts when there is an excess workload, adequate HH is missed,” some had given their opinion regarding missing their required compliance.

Almost all the nurses claimed that there had been no academic sessions for them regarding HH practices within at least previous six months, which could motivate them more in that regard.

“After patient contact, most of the time I rush into documenting the event in the file, without ensuring a required HH,”some doctors had expressed opinions on their practice regarding recommended HH after patient contact.

Almost all of the nurses claimed that repeated use of soap and hand rub solutions caused skin irritation. They wanted to have a bar of skin friendly liquid soap and adequately available hand moisturizer. Many of them wanted to have adequate sterile hand towels for wiping after HW along with hand dryers to save time.

Almost all of the doctors and nurses wanted to have regular academic sessions on importance of HH including the cleaners and other supporting staff of the NICU.

“Nurses and cleaners should be included in the ongoing infection control team,” they suggested.

Some of them wanted to have a monthly reward for the best compliance, which would be encouraging for HCWs.

## 4. Discussion

Adequate HH practice among the HCWs is one of the effective measures in preventing HCAI, especially in intensive care units, while poor compliance is associated with high rates of infections [8, 9].

Similar to the previous other studies, nurses were documented to have the highest number of patient contacts, 56.7% and 73.2% in the preintervention period and the postintervention period, respectively [15, 17, 19].

As documented in similar other studies, the emergency rescue contacts were excluded from analysis as prior HH actions were inadequate in all the events [18, 20].

In the preintervention period and the postintervention period, handwashing, hand rubbing, and missed hand hygiene opportunities were 27.9%, 36.2%, and 35.9% and 17.4%, 74.6%, and 8%, respectively. There was a significant increase in hand rubbing events (*p*=0.000243) and a significant decrease in missing events of HH (*p*=0.000) after the intervention. This observation goes in agreement with previous other studies [18, 21]. Pittet [22] observed improvement in HH compliance (48 to 66%) with the increase in use of alcohol-based hand rubs. A possible explanation was that nurses were unaware of the WHO recommendation of “ensuring prior HH even if gloves are used” [23], which was discussed in the group discussion sessions and relevant reminders were pasted in the NICU.

Use of gloves was indicated in 67.3% and 66.9% contacts in the preintervention period and the postintervention period, respectively. Of them, 48.6% contacts were not followed by prior HH in the preintervention period which was reduced significantly to 9.24% (*p*=<0.00001) in the postintervention period. Fuller et al. [12] and Ngugi et al. [24] described similar missing of prior HH in their studies. The incidence ofmissed wearing gloves (when it was indicated) was also significantly reduced (28% to 1.76%, *p*=<0.0001) after the intervention.

Following the intervention, a significant improvement was documented prior to both the high-risk and low-risk patient contacts, 43.1% vs. 84.9% (*p* < 0.000) and 43.1% vs. 81.8% (*p* < 0.000), respectively. Helder et al. [18] observed a similar significant improvement in compliance both in case of high-risk and low-risk contacts in their study.

During the preintervention period, the adequate and inadequate HH actions before patient contact were found 42.9% and 20.2%, respectively. Hand hygiene was missed before the rest of the 36.9% observations. Laskar et al. also observed as high as 47.2% inadequate HH actions. [14].

The proportions of incomplete and missed HH actions were decreased significantly (20.2 to 9.7%, *p*=0.00017 and 36.9 to 6.5%, *p*=<0.00001) after the intervention. Completeness of hand rubbing appeared to have improved notably with regard to in between the fingers, knuckles, fingertips, and thumbs. Similar significant reduction in the frequency of inadequate HH (*p*=0.01) and missing HH (*p*=0.01) was found in their study in the Netherlands by van den Hoogen et al. [19] Laskar et al. [14] had also observed a significant reduction in the proportion of incomplete HH action (47.2 to 21.4%, *p*=<0.00001), and missing HH (49.78–8.5%, *p*=<0.00001) after their intervention in a tertiary care center.

At the baseline observation, the overall compliance to HH was observed only 42.9% in this study.A similar finding of overall low compliance was observed in several other studies from Nepal, Kenya, Ghana, China, and Saudi Arabia, ranging from 9.2% to 41.1% [16, 17, 24–26]. In a review from 65 global studies, Eramus et al. documented an overall compliance rate of as low as 30–40% [27].

At the baseline observation, the proportion of HH compliance in this study was found 42.9% and 28.5% before and after patient contact, respectively. Compliance after patient contact was observed much less among doctors (15.6%) than nurses (38.4%). A possible explanation is rushing of doctors in the documentation of their activities immediately after patient contact. Compliance of HH after patient contact was significantly increased both in the case of doctors (15.6–97.8%, *p*=<0.00001) and nurses (38.4–95.2%, *p*=<0.00001) following the intervention. Anwar et al. observed a similar improvement in compliance of HH after patient contact (from 35% to 72.8%) in their study [28]. A significant improvement in HH compliance was also observed before patient contact (42.9 to 83.8%, *p*=<0.00001). The improvement was statistically significant both in the case of doctors and nurses (*p*=0.001125 and *p*=<0.00001).

Following an intervention, a similar improvement was observed in previous other studies as in Pondicherry, India (from 3 to 70.1%) [14], Nepal (from 9.2% to 68.8%) [25], Saudi Arabia (from 43% to 61.4%) [26], Egypt (30.9 to 69.5%) [28], Kuwait (60.8% to 86.4%) [29], Argentina (23.1% to 64.5%) [30], Brazil (30.0% to 56.7%) [31], and 51.0% to 67.2% in a multicenter multinational study including 43 hospitals in Costa Rica, Italy, Mali, Pakistan, and Saudi Arabia [32].

In the logistic regression analysis, HCWs were found significantly more compliant to ensure recommended HH after intervention (OR = 13.315, 95% CI: 7.248–24.458). Similarly, being a physician (OR = .012, 95% CI: 0.005–0.030) and events after patient contact (OR = 0.114, 95% CI: 0.049–0.261), significant determinants of compliance were found.

Similar risk factors for noncompliance were found in previous Saudi Arabian [26] and Egyptian studies [28]. Schweizer et al. [33] and Luangasanatip et al. [34] documented an education program as a predictor of increasing HH compliance OR: 1.47 and OR: 4.3, respectively in their studies.

As in previous other studies, the group discussion explored the reasons of noncompliance and way to increase compliance [35–38]. The most common causes of noncompliance were lack of regular relevant academic sessions, excessive workload, especially in the evening and night shifts, and skin damage from repeated HH actions. Similar to previous studies, the proposed ways in improving HH compliance were to ensure regular academic sessions on HH and pasting HH reminders in the NICU [36, 39, 40].

Proper hand hygiene before and after each contact with any patient is an effective and cost-efficient way to reduce the number of microorganisms, thereby reducing the rate of transfer of microorganisms to hospitalized patients and thus reducing the number of HCAIs. The result of this study showed that educational intervention, discussion sessions, and bedside reminders can significantly contribute in improving hand hygiene compliance.

One of the limitations of this study was that the analysis was conducted only on the HH opportunities before and after patient contact and no other HH opportunities. The rate of nosocomial infection and colonization in patients before and after the intervention was also not studied here.

## 5. Conclusion

Hand hygiene compliance among the HCWs was low. Prior hand hygiene was often missed when gloves were used. HH was frequently missing after patient contact. A significant improvement in HH compliance was achieved through an interventional approach. Placing reminder posters on HH in the strategic locations such as near hand wash sink, beside patient beds, and regular discussion on HH seemed to be most incisive for the positive attitude.

### 5.1. Recommendation

Regular arrangement of HH enhancing academic sessions, involving all the HCWs, posting reminders elsewhere in the workplace, and continuous surveillance of HH practices are recommended.

## Figures and Tables

**Table 1:** Frequency and characteristics of patient contacts.

Characteristics of HCWs	HH opportunity (*n*/*N*, %)	
	Preintervention	Postintervention
Job category		
Doctor	135/312 (43.3)	91/339 (26.8)
Nurse	177/312 (56.7)	248/339 (73.2)
Gender		
Male	53/312 (17)	29/339 (8.6)
Female	259/312 (83)	310/339 (91.4)
Category of patient contact		
Preplanned	312/340 (91.76)	339/361 (93.9)
Emergency	28/340 (8.24)	22/361 (6.1)
Diurnal period of observation		
Day time	227/312 (72.8)	146/339 (43.1)
Night time	85/312 (27.2)	193/339 (56.9)

**Table 2:** Changes in the pattern of hand hygiene action.

Pattern of HH	Observation period (*n*/*N*, %)		*χ* ^2^	*p* value
	Preintervention	Postintervention		
Hand wash (HW)	87/312 (27.9)	59/339 (17.4)		
Hand rub (HR)	113/312 (36.2)	253/339 (74.6)	13.4642	0.000243^s^
Missing prior HH action	112/312 (35.9)	27/339 (8.0)	75.4885	<0.001^s^

*s* = significant.

**Table 3:** Comparison of compliance to hand hygiene in patient contacts when gloves were used.

Use of gloves	Compliance to prior HH when the use of gloves was indicated (*n*/*N*, %)		*χ* ^2^, *p* value
	Preintervention	Postintervention	
Gloves with prior HH	49/210 (23.4)	202/227 (89)	192.3335, <0.001^s^
Gloves without prior HH (HH was missed when gloves were used)	102/210 (48.6)	21/227 (9.24)	93.1012, <0.001^s^
Missed wearing gloves but was indicated	59/210 (28)	4/227 (1.76)	61.3083, <0.001^s^

*s* = significant.

**Table 4:** Comparison of changes in hand hygiene compliance before and after intervention based on the risk category.

HH opportunity	Compliance (*n*/*N*, %)		*χ* ^2^, *p* value
	Preintervention	Postintervention	
High-risk contact	84/196 (42.9%)	185/218 (84.9)	80.0202, <0.001^s^
Low-risk contact	50/116 (43.1)	99/121 (81.8)	38.0255, <0.001^s^
Overall	134/312 (42.9%)	284/339 (83.8%)	117.842, <0.001^s^

*s* = significant.

**Table 5:** Changes in adequacy in HH action after intervention.

Category of HH compliance	Compliance (*n*/*N*, %)		*χ* ^2^, *p* value
	Preintervention	Postintervention	
Adequate HH	134/312 (42.9)	284/339 (84.8)	17.842, <0.001^s^
Inadequate HH	63/312 (20.2)	33/339 (9.7)	14.1336, <0.001^s^
No HH at all	115/312 (36.9)	22/339 (6.5)	90.1823, <0.001^s^

*s* = significant.

**Table 6:** Comparison of HH compliance before and after patient contact between doctors and nurses.

HH opportunity	Compliance (*n*/*N*, %)			
	Preintervention		Postintervention	
	Doctors	Nurses	Doctors	Nurses
Before patient contact	114/135 (84.4%)	20/177 (11.3%)	89/91 (97.8)	195/248 (78.6)
After patient contact	21/135 (15.6%)	68/177 (38.4%)	89/91 (97.8)	236/248 (95.2)

**Table 7:** Binary logistic regression analysis of potential factors determining hand hygiene noncompliance in the NICU.

Factors	aOR	95% CI		*p* value
		Lower	Upper	
Phase of the study: pre vs. postintervention	13.315	7.248	24.458	<0.001^s^
Job category: doctor or nurse	0.012	0.005	0.030	<0.001^s^
Events: after vs. before patient contact	0.114	0.049	0.261	<0.001^s^

## Data Availability

The data are available from the corresponding author upon request.
